# Identification of Genes Reveals the Mechanism of Cell Ferroptosis in Diabetic Nephropathy

**DOI:** 10.3389/fphys.2022.890566

**Published:** 2022-05-26

**Authors:** Xian Wang, Ling Jiang, Xue-Qi Liu, Yue-Bo Huang, Wei Zhu, Han-Xu Zeng, Li Gao, Li-Juan Ma, Meng-Ya Zhang, Qi-Jin Zhu, Yong-Gui Wu

**Affiliations:** ^1^ Department of Nephropathy, The First Affiliated Hospital of Anhui Medical University, Hefei, China; ^2^ Center for Scientific Research of Anhui Medical University, Hefei, China

**Keywords:** transcriptome, gene, diabetic nephropathy, glomerulus, ferroptosis, weighted gene co-expression network analysis

## Abstract

**Aims/Introduction:** Diabetic nephropathy (DN) is one of the main complications of diabetes. Genomics may reveal the essential pathogenesis of DN. We analyzed datasets to search for key genes to explore pathological mechanisms of DN.

**Materials and Methods:** In this study, weighted gene co-expression network analysis (WGCNA) was used to divide the differential expression genes (DEGs) from GSE142025 into different modules, and enrichment pathway analysis was conducted for each module to find key genes related to cell death pathway. Then, verification was carried out through network and histopathology. Finally, the regulatory mechanisms of key gene expression, including transcription factors (TFs), miRNA and E3 ligases related to ubiquitination, were predicted through website prediction and then miRNA results were validated using GSE51674 dataset.

**Results:** The results of WGCNA and enrichment pathway analysis indicated that ferroptosis had significantly occurred in advanced DN (AND) group. Analysis of DEGs indicated that the occurrence and development of ferroptosis are mainly through ALOX15-mediated lipid metabolism pathway, which was found in all intrinsic cells of the glomerulus detected by IHC and IF staining. Moreover, network predictions were used for searching ALOX15-related TFs and ubiquitination. Meanwhile, the network predictions combining with other dataset furtherly discovered miRNAs which regulated ALOX15 expression. This study showed that the levels of mmu-miR-142-3p increased in DN mice kidney tissues, compared with the NC group.

**Conclusion:** Ferroptosis existed in glomerular intrinsic cells of ADN group and its potential key candidate gene was ALOX15 which may be regulated by miR-142 and miRNA-650, TFs (CREBBP, EP300, HDAC1, MTA1, SPI1, STAT6) and E3 ligases related to ubiquitination (PML, ZMIZ1, MARCHF1, MARCHF3, MARCHF8, MARCHF11).

## Introduction

Diabetic nephropathy (DN) is one of the main complications of diabetes and the most important factor affecting the outcome of diabetes. The epidemiological investigation of chronic kidney disease (CKD) in China reported in 2016 suggested that CKD related to diabetes has become the most important cause of CKD in both the general population and a hospitalized urban population (Zhang et al., 2016). Therefore, the study of DN is of great significance.

The occurrence and development of DN is very complicated. A large number of cells and molecular mechanisms associated with the pathogenesis of DN have been reported, but the dominant role of which is still unknown. We may be able to find the key mechanism of DN by reversely deducing the regulatory factors of the major forms of cell death observed in DN, since the one of main cell outcome is cell deaths including apoptosis, pyroptosis, necroptosis, necrosis, ferroptosis. It is worth noting that “ferroptosis” named in 2012 (Dixon et al., 2012) is appreciated as one of the most widespread and ancient forms of cell death, because of the similarity to ferroptosis-like cell death observed in evolutionarily remote species, such as plants protozoa and so on (Distefano et al., 2017; Bogacz and Krauth-Siegel, 2018). Previous studies have observed the existence of ferroptosis in renal tubules in DN (Li et al., 2021; Wang et al., 2021), but which in glomerulus and its mechanism still need to be further explored.

Current studies have expounded the pathogenesis of DN from several aspects, such as total transcriptional genomics, proteomics, and metabolomics, with genomics being the earliest developed and the one with most available data. Genetic changes may reveal the essential pathogenesis of DN. However, it should be noted that DN is a chronic progressive disease, and there may be differences in the main mechanisms of the lesions in each stage of this disease, while many patients with DN in GEO were accompanied by large amounts of albuminuria and decreased renal function (Sircar et al., 2018; Song et al., 2019). At present, few reports studied the dynamic changes of gene expression during the onset and progression of diabetes mellitus. Weighted gene co-expression network analysis (WGCNA) is commonly used for analyzing high-throughput gene expression data with different characteristics, to mine gene co-expression networks and intramodular hub genes based on pairwise correlations in genomic applications. The advantage of this analysis method is that it can quickly screen gene sets related to specific samples or traits from datasets to find the core genes that play an important role in transcriptional regulation. There was a report about the RNA sequencing (RNA-seq) analysis of whole kidney biopsy samples of early and advanced DN patients (Fan et al., 2019), that focused on renal tubulointerstitial cells but did not include an in-depth analysis of the glomerulus, which is the key location of albuminuria formation. WGCNA analysis of this dataset may be able to identify key genes that are more closely connected with the progression of DN.

**FIGURE 1:**
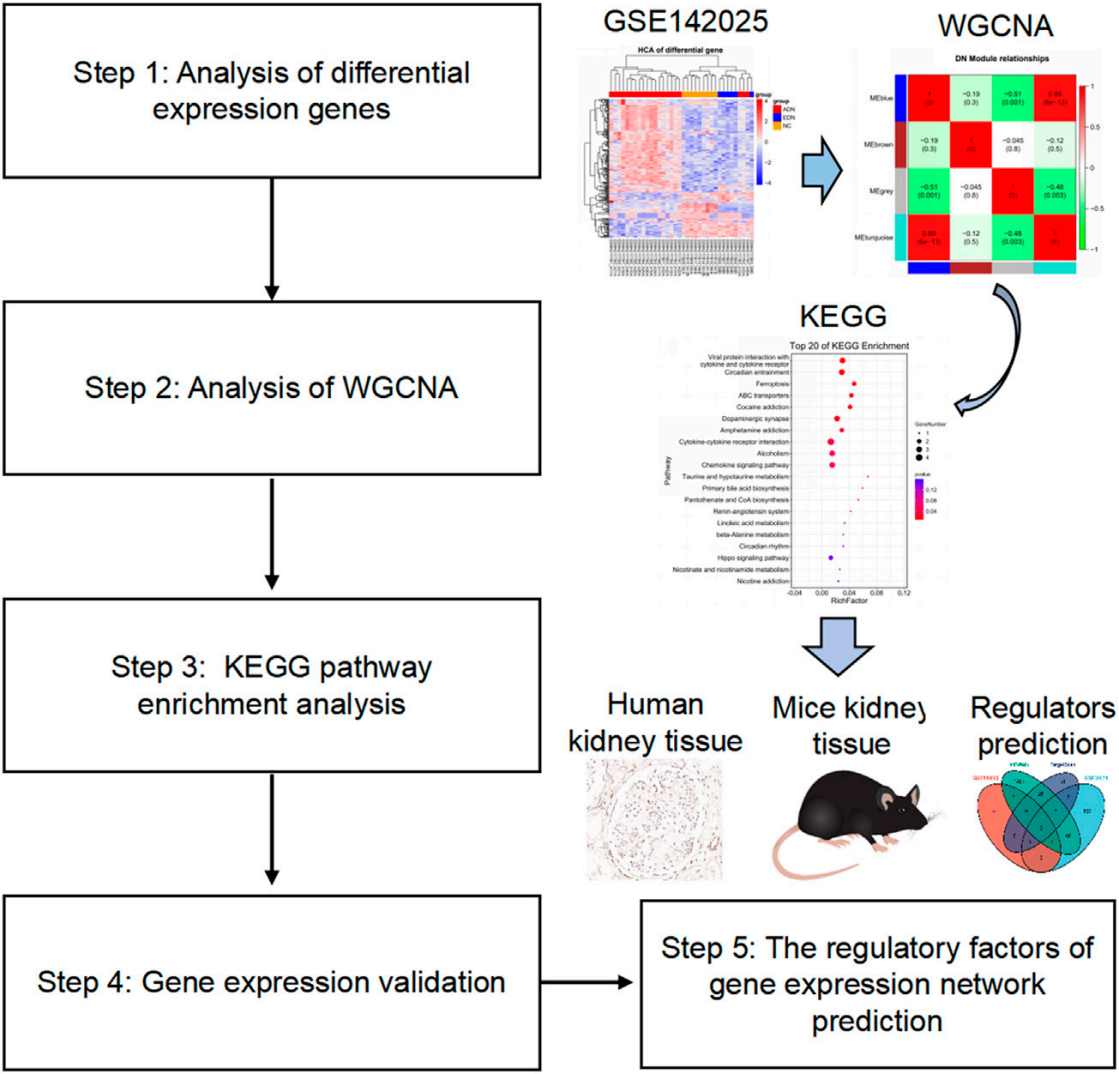
Flow chart of the study design. The raw data were obtained from the GEO database and analyzed using WGCNA. Then, KEGG enrichment analysis observed signaling pathways related to the occurrence and development of diabetic nephropathy. The validation of target gene expression and function were performed by detecting related genes and proteins in human and mouse kidney tissues. Finally, the regulatory factors of target genes were preliminarily explored through network prediction and real-time PCR experiments. GEO, gene expression omnibus; WGCNA, weighted gene co-expression network analysis; KEGG, Kyoto Encyclopedia of Genes and Genomes; PCR, polymerase chain reaction.

In this study, we searched for differential expression genes (DEGs) of GSE142025 accessed from the NCBI-Gene expression omnibus database (NCBI-GEO) to explore the main pathogenesis of DN from a cellular perspective by using WGCNA. Further, the expression, function and regulatory factors of target genes were observed and initially explored (Figure 1).

## Materials and Methods

### Data Collection and Preprocessing

The gene expression profile datasets of GSE142025 (Fan et al., 2019) were acquired from NCBI-GEO (http://www.ncbi.nlm.nih.gov/geo/) based on the GPL 20301 platform. A total of 36 patients were identified from the database, including six early DN patients (EDN group), 21 advanced DN patients (ADN group), nine control kidney tissue samples obtained from the unaffected portion of tumor nephrectomies (NC group), a case of absence in ADN group. The GSE51674 (Conserva et al., 2019) dataset was downloaded for subsequent analysis of miRNAs, which regulated the expression and function of target genes. Ferroptosis-related genes were acquired from FerrDb (http://www.zhounan.org/ferrdb/).

### Identification of Differential Expression Genes

The downloaded files from GSE142025 were processed by the R software package. Differential functions were analyzed using the Wilcox-test between the two groups. Genes with FDR < 0.05 and fold change ≥1.5 or fold change ≤0.67 were defined as DEGs.

### Gene Ontology and Kyoto Encyclopedia of Genes and Genomes Pathway Enrichment Analysis of Differential Expression Genes

In order to explore the functions and pathways involved in DEGs across each group, GO and KEGG enrichment of the DEGs were performed using the DAVID online tool (https://david.ncifcrf.gov). Both GO and KEGG showed the top 20 pathways.

### WGCNA Analysis of Differential Expression Genes

In the present study, we applied the WGCNA R package to analyze key gene clusters that were most relevant to ADN and EDN samples. The gene co-expression network needs to satisfy the scale-free network distribution: the logarithm [log(κ)] containing the number of nodes with a connection degree of κ is negatively correlated with the logarithm [log(P(κ))] of the probability of the occurrence of this node, and the correlation coefficient between the two is greater than 0.85. The higher the coefficient is, the more the network complies with the rules of a scale-free network, and for each module, the average connectivity of each gene should be high enough to ensure that the detection of such module is meaningful. In this study, the appropriate weighting coefficient β (soft threshold) was selected to transform the WGCN correlation matrix into a topological overlap matrix (TOM), and the dissimilarity as the distance measurement was used to divide samples into different genetic modules with different colors. Correlation scatter plots of modules and clinical groups were drawn respectively to specifically explore the correlation between each module and its corresponding group. To explore the correlation between the modules and the group information, a correlation heat map of the modules and their corresponding group information was drawn. A KEGG pathway enrichment analysis was performed for each module.

### Chemicals and Reagents

Streptozotocin (STZ) was purchased from Sigma-Aldrich (MO, United States). Anti-nephrin, anti-Pecam1(CD31), anti-PDGFR-β were acquired from Santa Cruz (Santa Cruz Biotechnology, Inc., United States). Anti-ALOX15 was acquired from Affinity Biosciences (Jiangsu, China) and Abcam Biotechnology (Cambridge, MA, United States). Anti-GPX4, secondary antibodies used for immunohistochemical and immunofluorescence staining were purchased from Proteintech Group, Inc. (Hubei, China). An immunohistochemistry kit (PV-6000) was acquired from Beijing Zhongshan Biotechnology Inc. (Beijing, China).

### Volunteers in This Study

A cross-sectional study of clinical patients was performed in this study. The total 12 patients with biopsy-proven DN hospitalized from January 2019 to July 2021 in the First Affiliated Hospital of Anhui Medical University were enrolled in the study. The inclusion-exclusion criteria were performed as previous described (Zhou et al., 2021). Fasting plasma glucose test (FPG) and estimated glomerular filtration rate (eGFR) were performed before renal biopsy puncture or surgery. DN patients were divided into two groups (*n* = 6/group) according to urinary albumin to creatinine ratio (UACR): early DN (EDN) (30–299 mg/g), advanced DN (ADN) (>300 mg/g). Six paracancer kidney samples without diabetes and abnormal changes of renal-related markers (eGFR values and urine routine examination) were used as the normal control group (NC group) for correlation analysis with the expression of taget genes and protein. Routine urine tests of NC group were negative. The clinical data and biopsy samples of volunteers were collected with the consent of the participants. This study was approved by the institutional review board of the First Affiliated Hospital of Anhui Medical University. All patients signed informed consent before participating in the study.

### Animal Models

The STZ-induced DN model was established using C57/J male mice aged 6–8 weeks, which were divided into two groups: The DN group (*n* = 6) and NC group (*n* = 6). The modeling method was processed as previously described (Liu et al., 2021). Mice in the two groups were kept in standard environment for 12 weeks after the success of the diabetes model. Animal models of type 2 diabetes were used *db/db* male mice (*n* = 6), while *db/m* male mice (*n* = 6) were used as the control group. All animals in both groups were fed for 20 weeks. After anesthesia, all mice underwent cardiac perfusion, and then kidney tissues were collected, part of which was placed at −80°C for subsequent PCR experiments.

### Immunohistochemical Analysis

Paraffin sections of the kidney tissue were dewaxed, repaired as previous (Liu et al., 2021), and then incubated with the primary antibody overnight at 4°C after antigen blocking with hydrogen peroxide and 10% BSA, followed by application of the secondary antibody at 37°C for 1 h. ImageJ software was used for IHC analysis.

### Immunofluorescence Analysis

The dewaxing and antigen recovery method of paraffin sections were the same as that used for IHC. These slices were incubated with the primary antibody at 4°C overnight after sealing with 10% BSA at 37°C for 1 h, followed by addition of the fluorescent secondary antibody (488 and 594 nm) at 37°C for 1 h. DAPI staining was performed at RT for 5 min. Finally, the slides were photographed using an inverted fluorescence microscope (Zeiss Spot; Carl Zeiss Ltd., Canada).

### RNA Isolation and Real-Time PCR

Total RNA was extracted and then reverse transcribed into cDNA as previously done (He et al., 2019; Liu et al., 2021). The cDNA was amplified using a T100 thermal cycler (Bio-Rad, Hercules, CA, United States). Real-time PCR experiments were performed using the ABI QuantStudio 5 (Applied Biosystems, Foster City, CA, United States) according to the manufacturer’s instructions. The β-actin gene and U6 snRNA were used as an endogenous control for mRNAs and miRNAs, respectively. The primers used for this assay are shown in Table 1.

**TABLE 1:** Primer sequences used in this study.

Primer	Forward primer	Reverse primer
ALOX15	GAC​ACT​TGG​TGG​CTG​AGG​TCT​T	TCT​CTG​AGA​TCA​GGT​CGC​TCC​T
β-actin	CAT​TGC​TGA​CAG​GAT​GCA​GAA​GG	TGC​TGG​AAG​GTG​GAC​AGT​GAG​G
mmu-miR-142-3p	GGT​GTA​GTG​TTT​CCT​ACT​TTA​TGG​A	ATC​CAG​TGC​AGG​GTC​CGA​GG
mmu-miR-142-3p Stem loop RT primer	GTC​GTA​TCC​AGT​GCA​GGG​TCC​GAG​GTA​TTC​GCA​CTG​GAT​ACG​ACT​CCA​TA	

### Single-Cell RNA Sequencing Reanalysis

The single-cell data was acquired from KIT (http://humphreyslab.com/SingleCell/) (Guo et al., 2021). The database was from 3 nondiabetic controls and 3 diabetics following nephrectomy for renal mass (Wilson et al., 2019).

### Target Gene Multi-Factor Regulatory Analysis

The prediction of the interactions between the target gene and miRNA dataset were performed by using the computational algorithms (Conserva et al., 2019): TargetScan 8.0 (http://www.targetscan.org/) and miRWalk (http://mirwalk.umm.uni-heidelberg.de/) to search the target gene-related miRNAs, which were then validated in key genes of GSE142025 and DEGs between NC and DN group from GSE51674 to acquired target miRNAs. Meanwhile, this study used TRRUST V2 (www.grnpedia.org/) database to predict transcription factor (TF)- Gene (protein) interaction (Hu et al., 2021). Additionally, ubibrowser 2.0 (http://ubibrowser.ncpsb.org.cn/) was used to observe proteins involved in the ubiquitination process of target proteins.

### Statistical Analysis

R 4.0.3 (https://www.r-project.org/) and SPSS 22.0 (IBM Corporation, Armonk, NY, United States) were used for our statistical analyses. The normality of the data was analyzed by the Shapiro-Wilk test and quantile–quantile plots. Categorical and no-normal distribution data was compared across groups using chi-square test or the rank sum test. Quantitative data were calculated as the mean ± SD. Statistical significances of the normal distribution data between groups were validated by independent-samples t test or single factor analysis of variance (ANOVA), followed by mapping with the GraphPad Prism 8 software (GraphPad Software Inc., San Diego, CA, United States). The statistical significance of non-normally distributed data was calculated by Mann-Whitney U test. The linear correlation analysis was using the Spearman test. *p* < 0.05 was considered the threshold for statistically significant difference between groups.

## Results

### Identification of Differential Expression Genes

**FIGURE 2:**
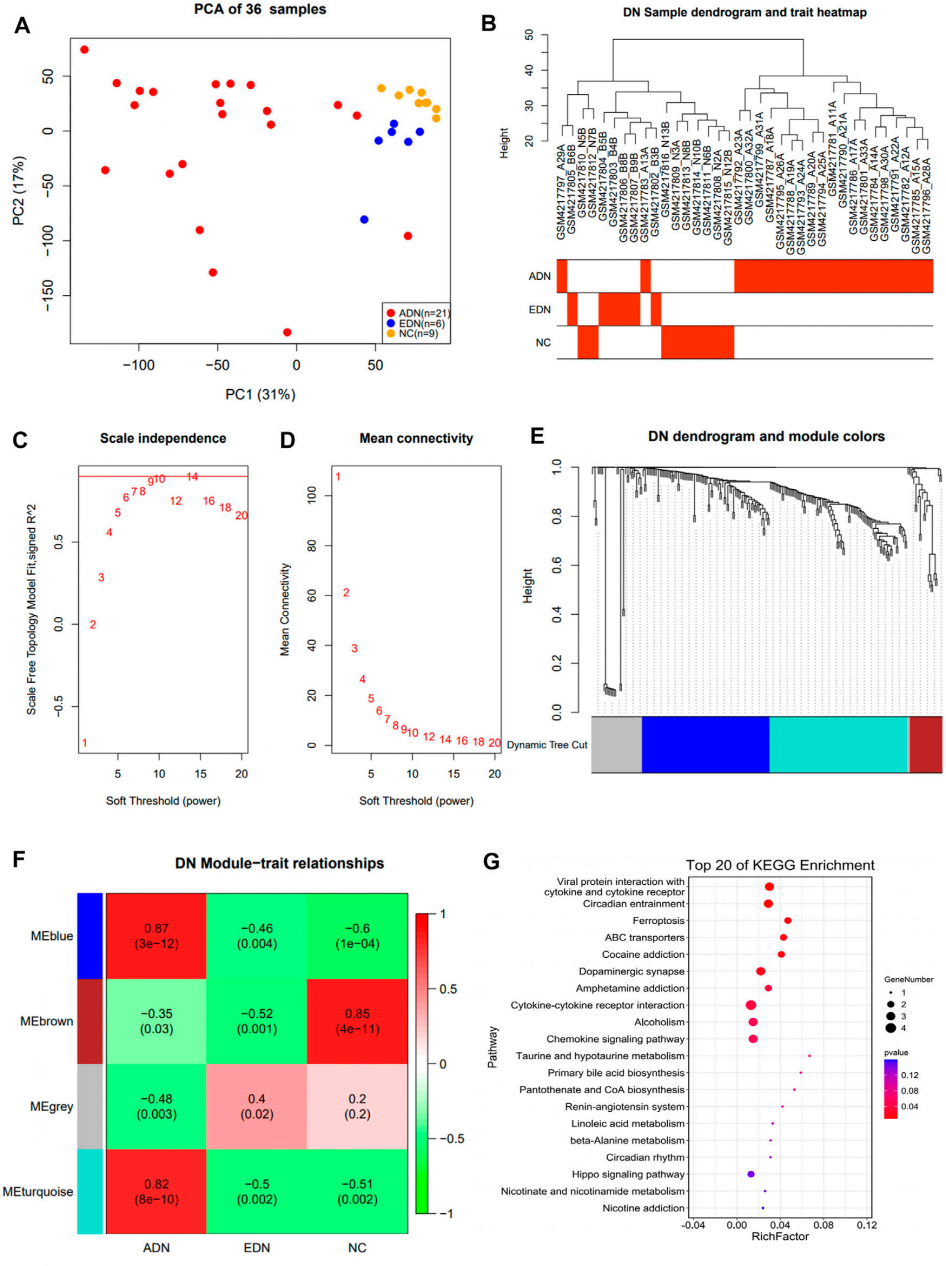
Co-expression network construction of GSE142025 dataset *via* WGCNA. **(A)** PCA clustering results of population samples. **(B)** DN sample dendrogram and trait heatmap. **(C)** Relationship between scale-free topology model and soft-thresholds. **(D)** Relationship between the mean connectivity and various soft-thresholds. **(E)** Dendrogram of DEGs. **(F)** Heatmap of the trait relationships between module genes and clinical traits of DN. **(G)** The top 20 significant KEGG pathways in blue module. WGCNA weighted gene co-expression network analysis; DN diabetic nephropathy; DEG differential expression genes; KEGG Kyoto Encyclopedia of Genes and Genomes.

The study identified 17,182 genes by comparing data from three groups of samples. Principal component analysis (PCA) indicated that 3 groups of samples were clustering together separately (Figure 2A). A total of 17,15,527 DEGs were identified among these three groups.

### Gene Ontology and KEGG Analysis of Differential Expression Genes

GO and KEGG enrichment was performed to explore the functions and pathways involved in DEGs among each group. The results showed that the pathways were mainly concentrated in cytokine-cytokine receptor interactions, lipid metabolism, inflammation and immune signaling pathways (Supplementary Figure S1A).

### WGCNA Network Construction and Signaling Pathway Enrichment Analysis

In this study, the data from 242 DEGs and 36 samples from three sets of differential analyses were used for WGCNA analysis. Heat maps of sample clustering and grouping information were drawn, and the specific distribution of samples in different groups were illustrated in Figure 2B. A scale-free network was built by 10 as the soft threshold (Figures 2C,D), which divided 242 differential genes into three modules with different colors (blue, turquoise, and brown). The Blue module contained 88 genes, the turquoise module contained 95 genes, and the brown module contained 22 genes. The clustering results among various modules revealed that blue and turquoise were closer to each other, while brown was farther from the other two modules (Figure 2E; Supplementary Figure S1B). Module correlation heat map suggested that blue and turquoise had a significantly positive correlation with a correlation coefficient of 0.89 (*P*<0.001) (Supplementary Figure S1C). The heat map of module and group information correlation indicated that there was a significant difference in correlation between each module and clinical group (*p* < 0.05). There was a significantly strong positive correlation between blue and turquoise modules and the ADN group (correlation coefficients of 0.87 and 0.82, respectively) (Figure 2F). A Scatter plot of correlation between modules and clinical groups indicated that the blue module was the most significant correlation with the three groups (Supplementary Figure S1C). KEGG pathway enrichment analysis suggested that the DEGs in three modules were primarily associated with cytokine-cytokine receptor interaction, inflammation, and immune cells related signaling pathways. Notably, the ferroptosis signaling pathway was evident in ADN (Figure 2G; Supplementary Figure S2).

### Detection of Ferroptosis in DN

**FIGURE 3:**
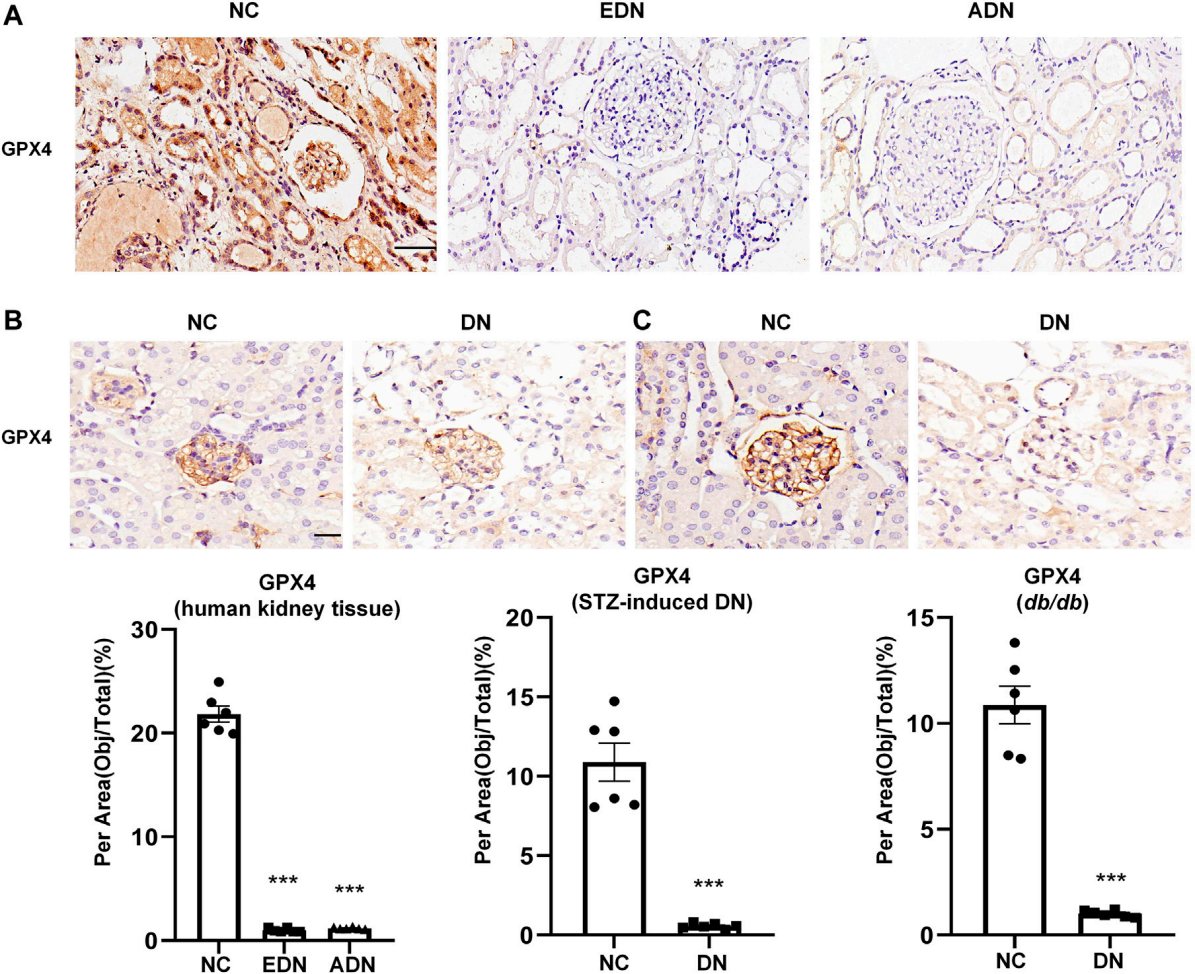
Detection of ferroptosis in DN **(A)** IHC assay of GPX4 protein expression on human renal biopsy tissues. Scale bar = 20 μm. **(B)** IHC assay of GPX4 protein expression on kidney tissues of STZ-induced diabetes mice models. Scale bar = 20 μm. **(C)** IHC assay of GPX4 protein expression on kidney tissues of *db/db* mice models. Scale bar = 20 μm. Results represented the mean ± SEM for six samples/group. ^***^ *p* < 0.001 vs. NC. NC normal control; DN diabetic nephropathy; EDN early DN; ADN advanced DN; STZ streptozotocin; IHC immunohistochemical staining.

This study examined changes in ferroptosis in DN by detecting glutathione peroxidase 4 (GPX4) protein expression, which is a recognized marker of ferroptosis. The results showed that GPX4 protein expression was observed in the glomerulus, tubules, and some interstitial cells, which decreased significantly after the occurrence of DN (Figure 3A). In STZ-induced diabetic mice (Figure 3B) and *db/db* mice diabetes models (Figure 3C), GPX4 protein expression in the DN group was also dramatically downregulated compared with the NC group.

### Ferroptosis Related DEGs Analysis

**FIGURE 4:**
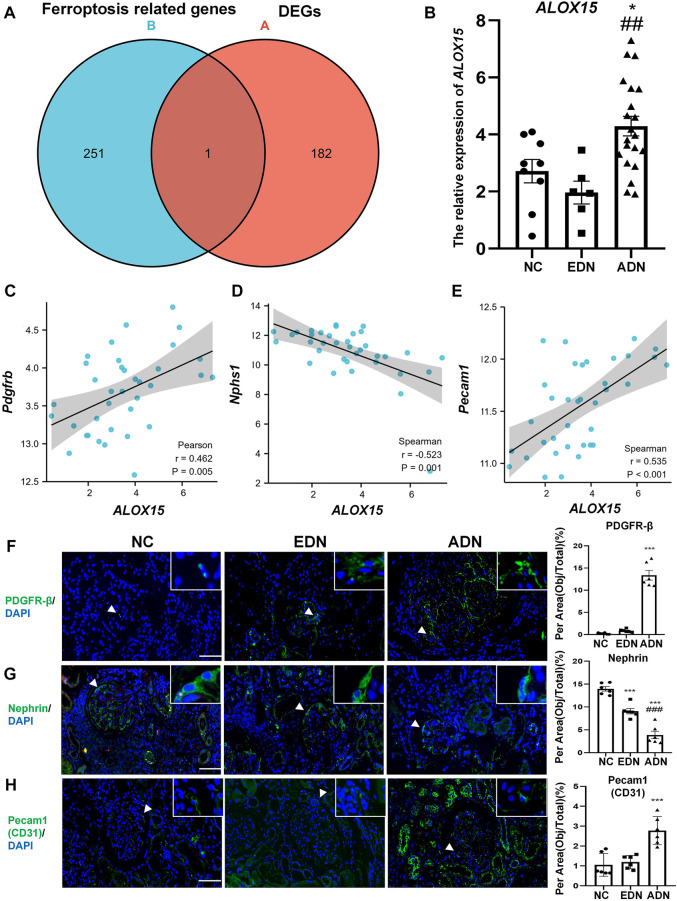
Ferroptosis related DEGs analysis. **(A)** The Venn map of common genes between GSE142025 and FerrDb. **(B)** The relative expression of *ALOX15* mRNA form GSE142025. **(C)** Correlation between *ALOX15* mRNA and the mRNA of mesangial cells marker (PDGFR-β) in GSE142025. **(D)** Correlation between *ALOX15* mRNA and the mRNA of podocytes marker (nephrin) in GSE142025. **(E)** Correlation between *ALOX15* mRNA and the mRNA of endothelial cells marker (pecam1/CD31) in GSE142025. **(F)** The protein expression of mesangial cells marker (PDGFR-β) in human kidney tissues of DN and NC groups using immunofluorescence double-staining analysis. Scale bar = 20 μm. **(G)** The protein expression of podocytes marker (nephrin) in human kidney tissues of DN and NC groups using immunofluorescence double-staining analysis. Scale bar = 20 μm. **(H)** The protein expression of endothelial cells marker (pecam1/CD31) in human kidney tissues of DN and NC groups using immunofluorescence double-staining analysis. Scale bar = 20 μm. Results represented the mean ± SEM for six samples/group. ^*^ *p* < 0.05, ^***^ *p* < 0.001 vs. NC; ^##^ *p* < 0.01, ^###^ *p* < 0.001 vs. EDN. DEGs differential expression genes; NC normal control; DN diabetic nephropathy; EDN early DN; ADN advanced DN.

We compared ferroptosis-related genes from the network and DEGs in blue and turquoise modules. Arachidonate 15-lipoxygenase (ALOX15) was only one of DEGs associated with ferroptosis (Figure 4A). The results of gene expression in the dataset demonstrated that the level of ALOX15 gene expression decreased slightly in EDN (not statistically significant), but increased significantly in the ADN group, which was positively correlated with endothelial and mesangial cell marker gene expression, and negatively correlated with podocyte marker expression (Figures 4B–E). The changes of cell markers in human DN were further observed. The results suggested that the levels of nephrin protein expression decreased gradually with the progression of the disease, while CD31 and PDGFR-β protein levels showed an opposite trend (Figures 4F–H).

### Expression of ALOX15 Gene and Protein in DN

**FIGURE 5:**
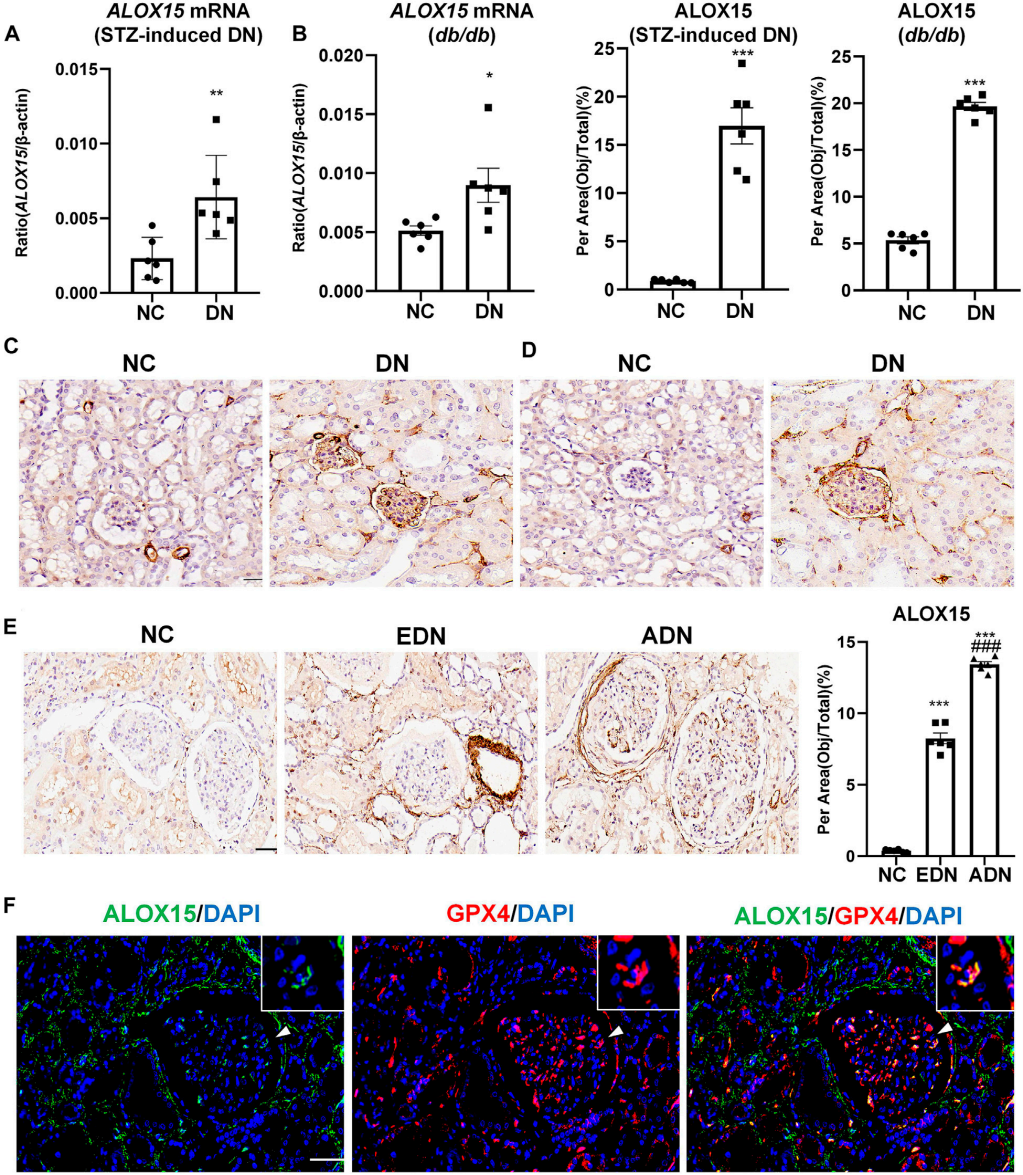
Expression of ALOX15 mRNA and protein in DN. **(A)** Real-time PCR assay of *ALOX15* mRNA in STZ-induced diabetes mice models. **(B)** Real-time PCR assay of *ALOX15* mRNA in *db/db* mice models. **(C)** IHC assay of ALOX15 protein expression on kidney tissues of STZ-induced diabetic mice models. Scale bar = 20 μm. **(D)** IHC assay of ALOX15 protein expression on kidney tissues of *db/db* mice models. Scale bar = 20 μm. **(E)** IHC assay of ALOX15 protein expression on human renal biopsy tissues. Scale bar = 20 μm. **(F)** Immunofluorescence double staining of GPX4 and ALOX15 protein co-expression on mice glomerulus. Scale bar = 20 μm. Results represented the mean ± SEM for six samples/group. ^*^ *p* < 0.05, ^**^ *p* < 0.01, ^***^ *p* < 0.001 vs. NC; ^###^ *p* < 0.001 vs. EDN. NC normal control; DN diabetic nephropathy; EDN early DN; ADN advanced DN; STZ streptozotocin; IHC immunohistochemical staining.

The results confirmed that ALOX15 mRNA level in the STZ-induced mice model group was significantly increased compared with the NC group, which was consistent with the results of mRNA detection in *db/db* mice (Figures 5A,B). IHC was used to preliminarily locate and for semi-quantitation of ALOX15 protein expression. In the animal model, ALOX15 protein expression in the DN group was significantly higher than that in the NC group (Figures 5C,D). The measurement results of human kidney tissue showed that compared with NC groups, ALOX15 protein expression was also markedly higher in the diabetic group, mainly in the glomerular and renal interstitium (Figure 5E). Immunofluorescence double staining suggested that GPX4 and ALOX15 were expressed in the same region, confirming that ALOX15 was an important factor involved in the occurrence and development of ferroptosis (Figure 5F).

### Correlation Analysis of ALOX15 Protein Expression and Pathological Indicators of DN Patients

**FIGURE 6:**
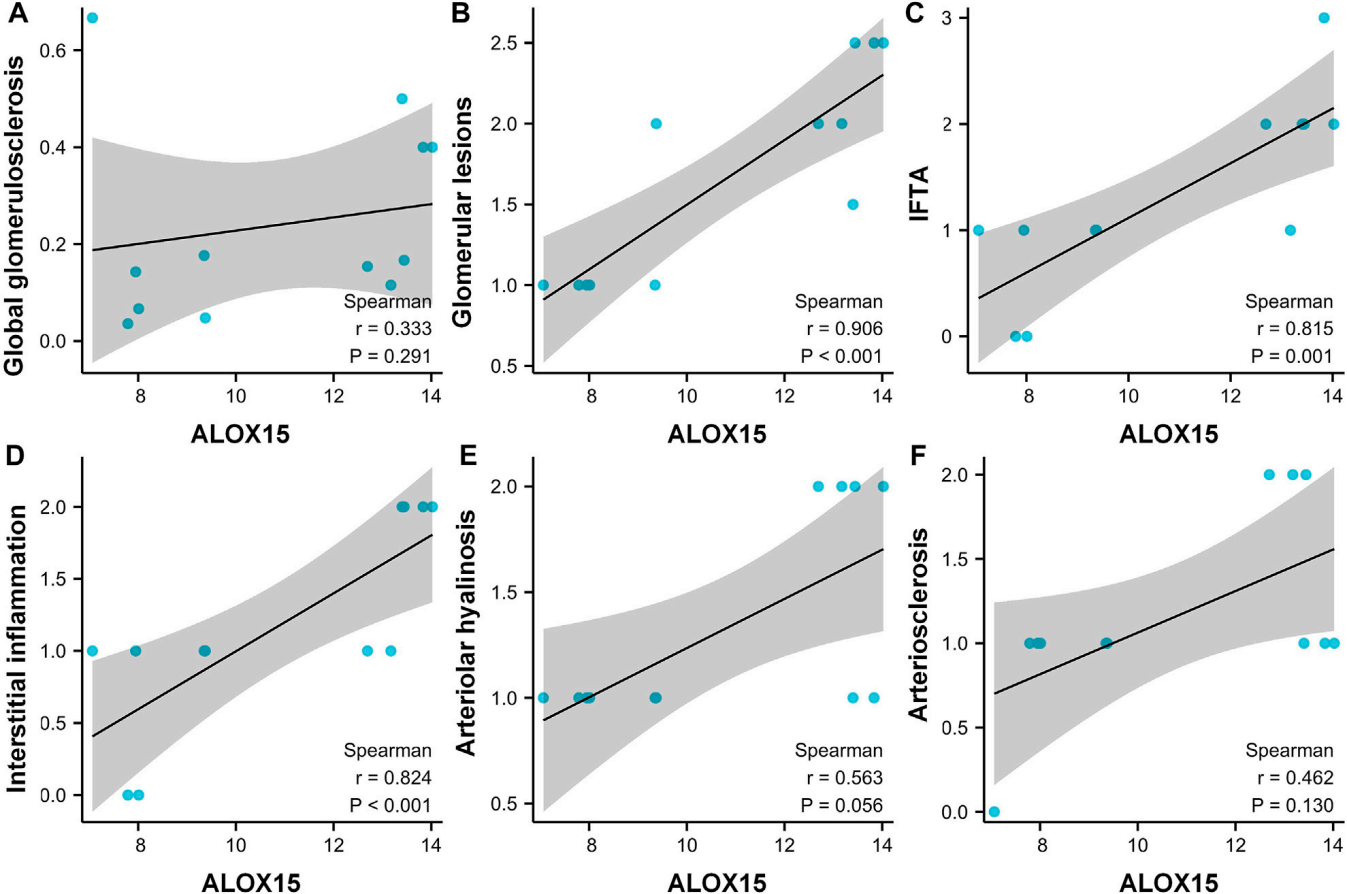
Correlation analysis between the expression of ALOX15 protein and the patient’s pathological characteristics. Correlations between the expression of ALOX15 protein and globalglomerulosclerosis **(A)**, glomerularlesions **(B)**, IFTA **(C)**, interstitialinflammation **(D)**, arteriolarhyalinosis **(E)** and arteriosclerosis **(F)**. Six samples per group were used for analysis. IFTA interstitial fibrosis and tubular atrophy.

The general characteristics of all Volunteers were delineated in Table 2, and pathological characteristics of DN patients were laid out in the Supplementary Table S1. The results denoted that the pathological features were significantly different between the two DN subgroups in global glomerulosclerosis rate, glomerular lesions, interstitial fibrosis and tubular atrophy (IFTA), interstitial inflammation, arteriolar hyalinosis, and arteriosclerosis. We then conducted a correlation analysis between the expression of ALOX15 and the patient’s pathological characteristics (Figure 6). The results suggested that changes of ALOX15 protein expression were significant correlations with various renal pathological changes, including glomerular lesions, IFTA, interstitial inflammation, but no correlation with global glomerulosclerosis, arteriolar hyalinosis and arteriosclerosis.

**TABLE 2:** General characteristics of all volunteers.

Characteristics	NC (*n = 6*)	EDN (*n = 6*)	ADN (*n = 6*)	*p* value
Age (years)	50.83 ± 5.57	42.50 ± 11.68	45.33 ± 9.11	0.261
Sex (male/female)	4/2	3/3	3/3	0.809
UACR (mg/g)	—	58.39 ± 44.21	3,417.00 ± 2,376.79^*^	0.018
FPG (mmol/L)	4.51 ± 0.88	4.85 ± 1.03	10.10 ± 4.83^**##^	

Values are means ± SD; *n* =6/group. NC vs. ADN *p < 0.05; EDN vs. AND ^##^ *p* < 0.01. NC, nomal control; EDN, early DN; ADN, advanced DN; UACR, urinary albumin excretion rate.

### Cellular Localization of ALOX15 Protein

**FIGURE 7:**
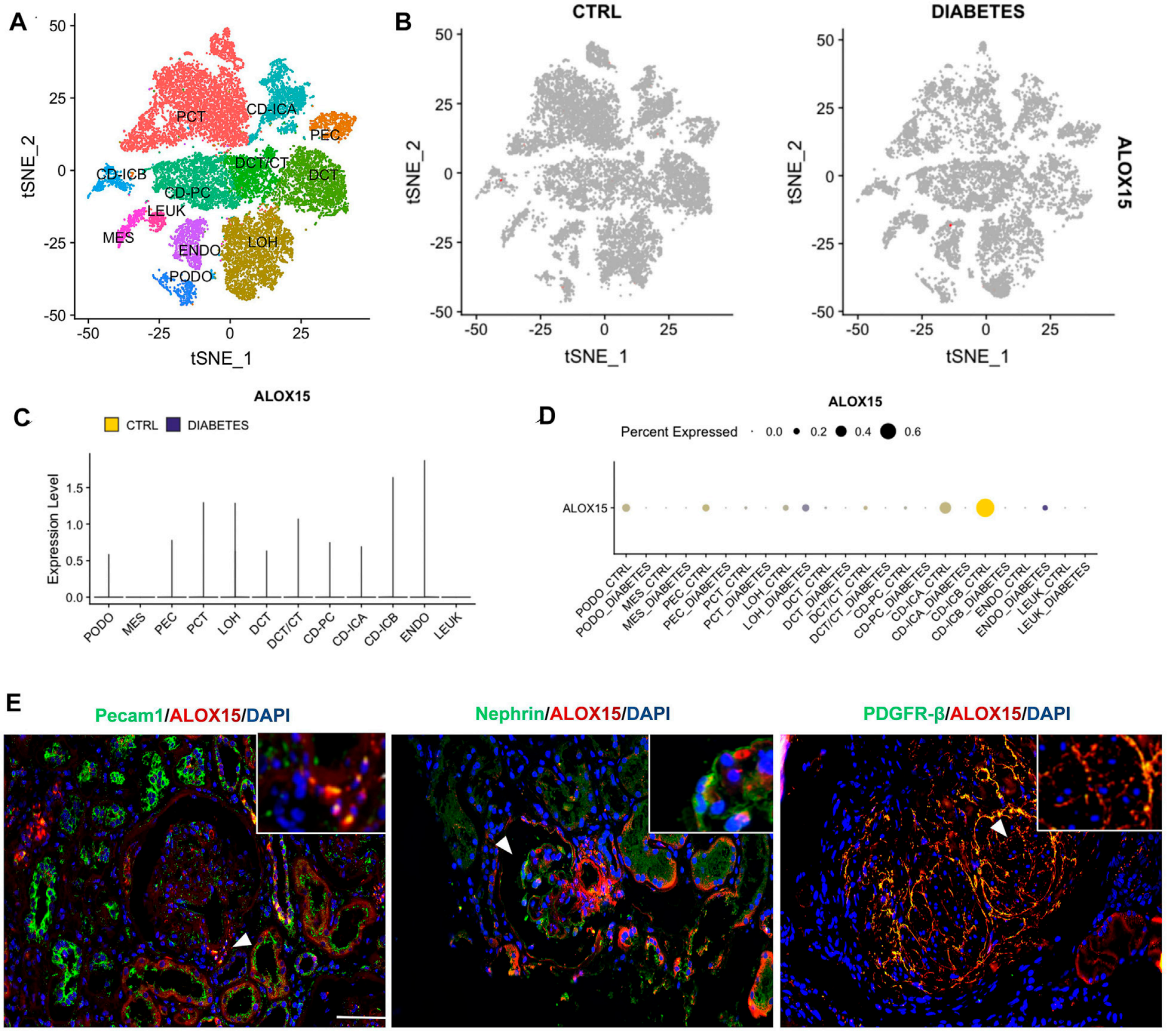
Cellular localization of ALOX15 protein expression. **(A)** A single dataset including DN and control samples. **(B)** The distribution of ALOX15 in different cells between healthy controls and DN. **(C)** The relative expression level of ALOX15 in identified cell types between healthy controls and DN. **(D)** The percent expression of ALOX15 in identified cell types between healthy controls and DN. **(E)** Immunofluorescence double staining of ALOX15 protein and cell markers co-expression on human kidney tissues of AND group. Scale bar = 20 μm. DN diabetic nephropathy; ADN advanced DN.

In this study, we focused on ALOX15 expression in intrinsic cells of the glomerulus, including podocytes, mesangial cells and endothelial cells. The analysis of single-cell sequencing data in KIT suggested that the renal cortex contains 11 types of cells, and the expression of ALOX15 in DN was mainly increased in glomerular endothelial cells (Figures 7A–D). However, immunofluorescence double staining was used to conduct a cell localization analysis of ALOX15 expression in the glomerulus. The results indicated that ALOX15 was expressed in mesangial cells, podocytes and endothelial cells (Figure 7E).

### ALOX15 Multi-Factor Regulation

**FIGURE 8:**
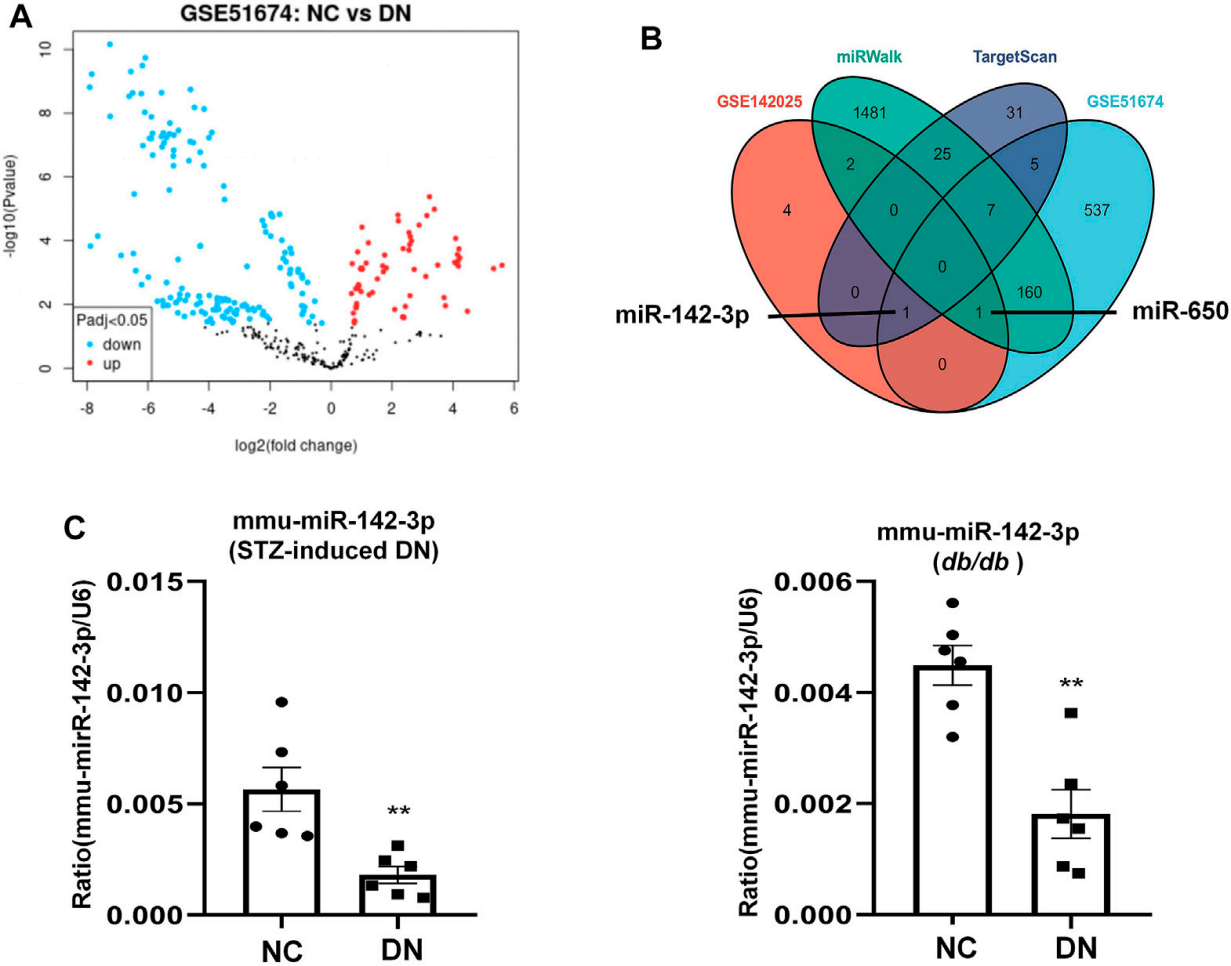
miRNAs regulate *ALOX15*. **(A)** The volcano plot of differentially expressed miRNAs. **(B)** The Venn map of common miRNAs between dataset and network prediction. **(C)** The levels of mmu-miR-142-3p in kidney tissues of STZ-induced DN and *db/db* mice models. Results represent the mean ± SEM for six samples/group. ^**^ *p* < 0.01 vs. NC. NC normal control; DN diabetic nephropathy; DEG differential expression genes; STZ streptozotocin.

TF and miRNA play major roles in regulating the synthesis and function of protein-coding genes. There were 8 differentiated miRNAs in GSE142025, including miR-6883, miR-3189, miR-5690, miR-650, miR-142, miR-4537, miR-4539, and miR-6863. Comparative analysis of the NC and DN groups in GSE51674 obtained 711 differential miRNAs (Figure 8A), With 69 hsa-miRNAs regulating ALOX15 predicted by TargetScanHuman 8.0, while the miRWalk website predicted 1,679 has-miRNAs related to ALOX15. The Venn diagram of genes showed that hsa-miR-142 and has-miR-650 were the target miRNAs regulating ALOX15 (Figure 8B). This study further examined miRNAs levels in a mouse model of DN. It was found that only hsa-miR-142-3p had corresponding mmu-miR-142-3p, through the search of mouse miRNAs corresponding to human miRNAs on the website (https://www.mirbase.org/). The results suggested that in the kidney tissues of the model group, the levels of mmu-miR-142-3p were significantly decreased compared with that in the NC group (Figure 8C). Meanwhile, we also predicted six ALOX15-related TFs: CREBBP, EP300, HDAC1, MTA1, SPI1, STAT6, and six E3 ligases related to the ubiquitination of ALOX15 proteins namely PML, ZMIZ1, MARCHF1, MARCHF3, MARCHF8, MARCHF11.

## Discussion

Recently, a large number of articles explored the pathogenesis of DN from multiple perspectives, one of the earliest and most common of the measures is the observation of genetic changes to find high specificity and sensitivity markers and therapeutic targets in the development and progression of DN.

However, previous samples were mostly limited to a certain time point of the disease, and there were few studies providing a dynamic observation of DN progression. The results reported a stronger correlation between blue and turquoise modules, and the correlation coefficient between the two modules and group was the highest, while the correlation between EDN and the three modules was not significant. Therefore, we focused on the analysis of DEGs in blue and turquoise modules. The KEGG enrichment analysis of DEGs in the two modules suggested that ferroptosis was significant in DN.

Ferroptosis is an iron-dependent form of regulated cell-programmed necrosis whose occurrence requires the accumulation of redox-active iron and cellular reactive oxygen species (ROS) and the peroxidation of phospholipid-containing polyunsaturated fatty acids, which are highly present in cell lipid membranes (Gao et al., 2015; Gao et al., 2016). The classic signal pathway of ferroptosis is GPX4-mediated lipid peroxidation resulting in cytotoxic effects (Ayala et al., 2014). It was confirmed that ferroptosis can be induced by compounds including erastin, ras-selective lethal small molecular 3 (RSL3), lanperisone, buthionine (Angeli et al., 2017; Latunde-Dada, 2017). Cell ferroptosis in DN has been reported recently. It has been reported that an increased level of ferroptosis aggravated DN and renal tubular injury, while fenofibrate inhibited ferroptosis by upregulating the level of NFE2-related factor 2 (Nrf2) expression, thereby reducing renal tubular injury and improving diabetic kidney injury (Wang et al., 2020; Li et al., 2021). Another report suggested that Sp1-mediated upregulation of Prdx6 expression mitigated oxidative stress and ferroptosis to prevent podocyte injury in DN (Zhang et al., 2021). This study also confirmed that GPX4 decreased significantly in the glomerulus and tubulointerstitial lesions, indicating that ferroptosis may occur in various cells with the development of DN. Interestingly, the ferroptosis pathway was more prominent in the ADN group, suggesting that cell ferroptosis may occur mainly in the middle and late stages rather than in the early stage of DN. Further research is needed to confirm this hypothesis.

Notably, unrestrained lipid peroxidation is the hallmark of ferroptosis. A part of lipoxygenases (LOXs), which are non-heme iron-dependent dioxygenases, can directly oxygenate polyunsaturated fatty acids (PUFAs) of biological membranes (Kuhn et al., 2015). There was a report that arachidonate-12-Lipoxygenase (ALOX12), was essential for p53-dependent ferroptosis, and the form of ferroptosis was independent of acyl-CoA synthetase long-chain family member 4 (ACSL4) (Chu et al., 2019). In this study, the DEG closely related to ferroptosis was ALOX15, which is also a member of the LOXs family. The 12/15-LOX enzymes react with polyunsaturated fatty acids producing active lipid metabolites that are involved in diabetic complications, such as neurological disorders (Li et al., 2019), retinopathy (Elmasry et al., 2018), while the 12/15-LOX pathway inhibitor was found to attenuate the kidney histopathological changes and renal injury induced by diabetes (Gad, 2012). The effect of 12/15-LOX on cell death initially focused on apoptosis, but recently a large number of literature reported that 12/15-LOX could affect autophagy and ferroptosis (Faulkner et al., 2015; Li et al., 2018). A study reported that the selective 12/15-LOX inhibitor and the pan-LOX inhibitor nordihydroguaiaretic acid protected acute lymphoblastic leukemia (ALL) cells against RLS3-induced ferroptosis (Probst et al., 2017). Another study showed that *ALOX15* silencing protected ferroptosis induced by erastin and RSL3 in oncogenic Ras-expressing cancer cells including HT1080 (human fibrosarcoma cell), PANC-1 (human pancreatic carcinoma cell) and CALU-1 (human non-small lung cancer). IF analyses revealed that the ALOX15 protein was consistently localized to the cell membrane in the process of ferroptosis (Shintoku et al., 2017). Previous reports have shown that the activation of 12/15-LOX resulted from reduced GSH, which is the key marker of ferroptosis (Li et al., 1997).

This study confirmed that the level of ALOX15 gradually increased with the progression of DN, through observation of the changes of *ALOX15* mRNA levels of DN mice and ALOX15 protein expression in the kidney tissue of human biopsy and mice models. Consequently, the above results verified that ALOX15 is closely associated with ferroptosis in DN. Cell localization of ALOX15 protein detected by immunofluorescence double staining confirmed that ALOX15 was significantly expressed in endothelial cells, podocytes and mesangial cells. Therefore, it was stipulated that ALOX15-mediated ferroptosis in the glomerulus occurred mainly in mesangial cells, podocytes and endothelial cells in DN.

Diabetic mesangial cell injury can be improved by activation of autophagy via miRNA-142-5p/PTEN signaling (Chen et al., 2019), while miR-142-3p has been reported to ameliorate high blood glucose-induced renal tubular epithelial cell injury (Zhao et al., 2021). In this study, mmu-miR-142-3p increased in DN mice kidney tissues, compared with the NC group. This suggested that miRNA may be a main regulator of ALOX15 mRNA. However, the relationship between miR-650 and ALOX15 in DN has not been reported. Therefore, the specific mechanism of ALOX15 mRNA regulators still needs to be further verified. Additionally, network prediction was used to obtain E3 ligases of ubiquitination and TFs, which regulated ALOX15 expression and degradation. The above factors and DEGs are collaboratively involved in the occurrence and development of DN and might be potential therapeutic targets.

In this study, DEGs enrichment pathway analysis, network prediction, histopathology and other dataset validation methods were used to confirm that ferroptosis was significantly present in DN and mainly related to lipid metabolism, and predicted the miRNAs, TFs, and ubiquitinated proteins involved in regulating ferroptosis. However, this study still has some limitations, such as the small sample size and the limited human transcriptome data. Additionally, it was notable that there was the lack of validation of ALOX15 mRNA levels and miR-142-3p in human tissues in this study, due to the limitation of practical conditions. Therefore, the genes, proteins, and regulatory mechanisms related to ferroptosis need to be further verified.

In conclusion, through integrated bioinformatics analysis, we have observed that ALOX15 is the potential key candidate gene involved in ferroptosis, which is mostly present in glomerular intrinsic cells of ADN, and is regulated by miRNAs (miR-142, miR-650), TFs (CREBBP, EP300, HDAC1, MTA1, SPI1, STAT6) and E3 ligases related to ubiquitination (PML, ZMIZ1, MARCHF1, MARCHF3, MARCHF8, MARCHF11). Furthermore, the results of this study promote further research on the molecular mechanisms underlying the pathogenesis and progression of DN, which will enable to identify therapeutic targets for DN in the future.

## Data Availability

Publicly available datasets were analyzed in this study. This data can be found here: http://www.ncbi.nlm.nih.gov/geo/, GSE142025; GSE51674.

## Supplementary Material

Supplementary Figure S1 The functional analysis of DEGs in GSE142025 dataset. **(A)** The top 20 significant KEGG pathways in ADN vs. EDN **(a)**, ADN vs. NC **(b)**, EDN vs. NC **(c)**, respectively. **(B)** Clustering of module eigengenes. **(C)** Relationship between module membership and gene significance in ADN group. DEG differential expression gene; KEGG kyoto Encyclopedia of Genes and Genomes; ADN advanced DN; EDN early DN; NC normal control.

Supplementary Figure S2 The functional analysis of DEGs in each module. The top 20 significant KEGG pathways in brown **(A)** and turquoise **(B)** module. DEG differential expression gene; KEGG kyoto encyclopedia of genes and genomes.

Supplementary Figure S3 Negative control of renal tissue staining. **(A)** Negative control of renal tissue immunofluorescent double staining. Scale bar = 50 μm. **(B,C)** Negative control of mice and human renal tissue staining by IHC, respectively. Scale bar = 50 μm.

## Abbreviations

ADN, advanced DN; ALOX15, arachidonate 15-lipoxygenase; ALOX12, arachidonate-12-Lipoxygenase; ACSL4, acyl-CoA synthetase long chain family member 4; ALL, acute lymphoblastic leukemia; ANOVA, analysis of variance; CKD, chronic kidney disease; DN, diabetic nephropathy; DEGs, differential expression genes; EDN, early DN; eGFR, estimated glomerular filtration rate; FPG, fasting plasma glucose test; GO, gene ontology; GPX4, glutathione peroxidase 4; IHC, immunohistochemical analysis; IF, immunofluorescence analysis; IFTA, interstitial fibrosis and tubular atrophy; KEGG, kyoto encyclopedia of genes and genomes; LOXs, lipoxygenases; MCD, minimal change disease; NCBI-GEO, NCBI-gene expression omnibus database; Nrf2, NFE2-related factor 2; PCA, principal component analysis; ROS, reactive oxygen species; RSL3, ras-selective lethal small molecular 3; STZ, streptozotocin; TOM, topological overlap matrix; TF, transcription factor; UACR, urinary albumin to creatinine ratio; WGCNA, weighted gene co-expression network analysis.
