# Supplementary material for: Identification of Genes Reveals the Mechanism of Cell Ferroptosis in Diabetic Nephropathy
Source: Front Physiol. 2022 May 26;13:890566. doi: 10.3389/fphys.2022.890566 (PMC9204496; doi:10.3389/fphys.2022.890566)
Supplement: Supplementary file 1 [file DataSheet1.ZIP › supple. Image and table/supple table.docx]

| **Characteristics** | **EDN（n=6）** | **ADN（n=6）** | **t/[Z]** | ***p* value** |
| --- | --- | --- | --- | --- |
| **Global glomerulosclerosis**  **rate（%）** | 8.933±5.675 | 28.932±16.281 | 2.841 | 0.029 |
| **Glomerular lesions** |  |  | 2.211 | 0.027 |
| **Class Ⅰ** | 0 | 0 |  |  |
| **Class Ⅱa** | 5 | 1 |  |  |
| **Class Ⅱb** | 0 | 0 |  |  |
| **Class Ⅲ** | 1 | 5 |  |  |
| **Class Ⅳ** | 0 | 0 |  |  |
| **IFTA** |  |  | 2.713 | 0.007 |
| **0** | 2 | 0 |  |  |
| **1** | 4 | 1 |  |  |
| **2** | 0 | 4 |  |  |
| **3** | 0 | 1 |  |  |
| **Interstitial infammation** |  |  | 2.447 | 0.014 |
| **0** | 2 | 0 |  |  |
| **1** | 4 | 2 |  |  |
| **2** | 0 | 4 |  |  |
| **Arteriolar hyalinosis** |  |  | 2.345 | 0.019 |
| **0** | 0 | 0 |  |  |
| **1** | 6 | 2 |  |  |
| **2** | 0 | 4 |  |  |
| **Arteriosclerosis** |  |  | 2.021 | 0.043 |
| **0** | 1 | 0 |  |  |
| **1** | 5 | 3 |  |  |
| **2** | 0 | 3 |  |  |

**Supple table 1 Renal pathological characteristics of DN patients**

Data are expressed as number

IFTA interstitial fibrosis and tubular atrophy.
