# Supplementary figures and images for: Identification of Genes Reveals the Mechanism of Cell Ferroptosis in Diabetic Nephropathy
Source: Front Physiol. 2022 May 26;13:890566. doi: 10.3389/fphys.2022.890566 (PMC9204496; doi:10.3389/fphys.2022.890566)

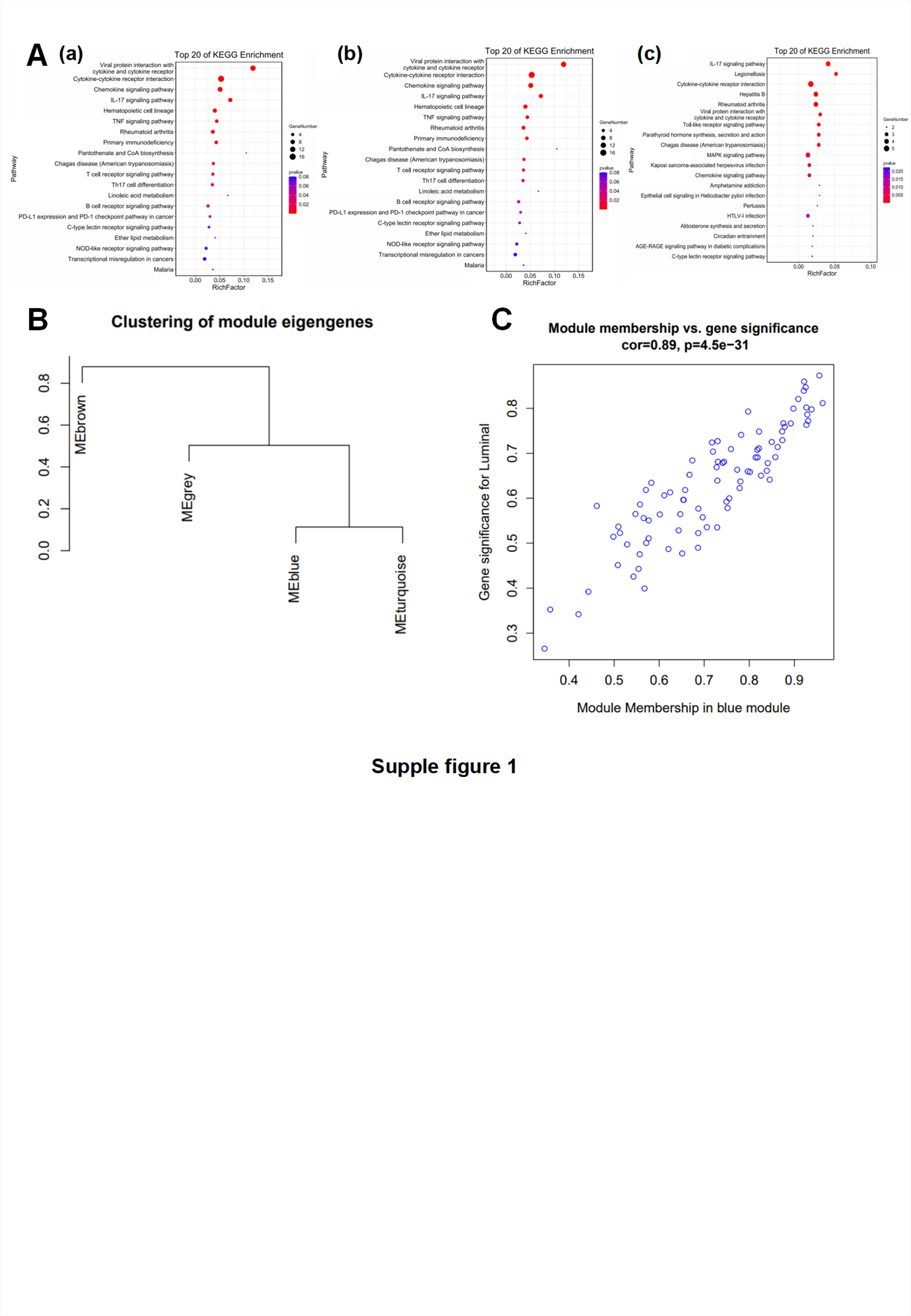

Supplement: Supplementary file 1 [file DataSheet1.ZIP › supple. Image and table/supple figure 1.tif]

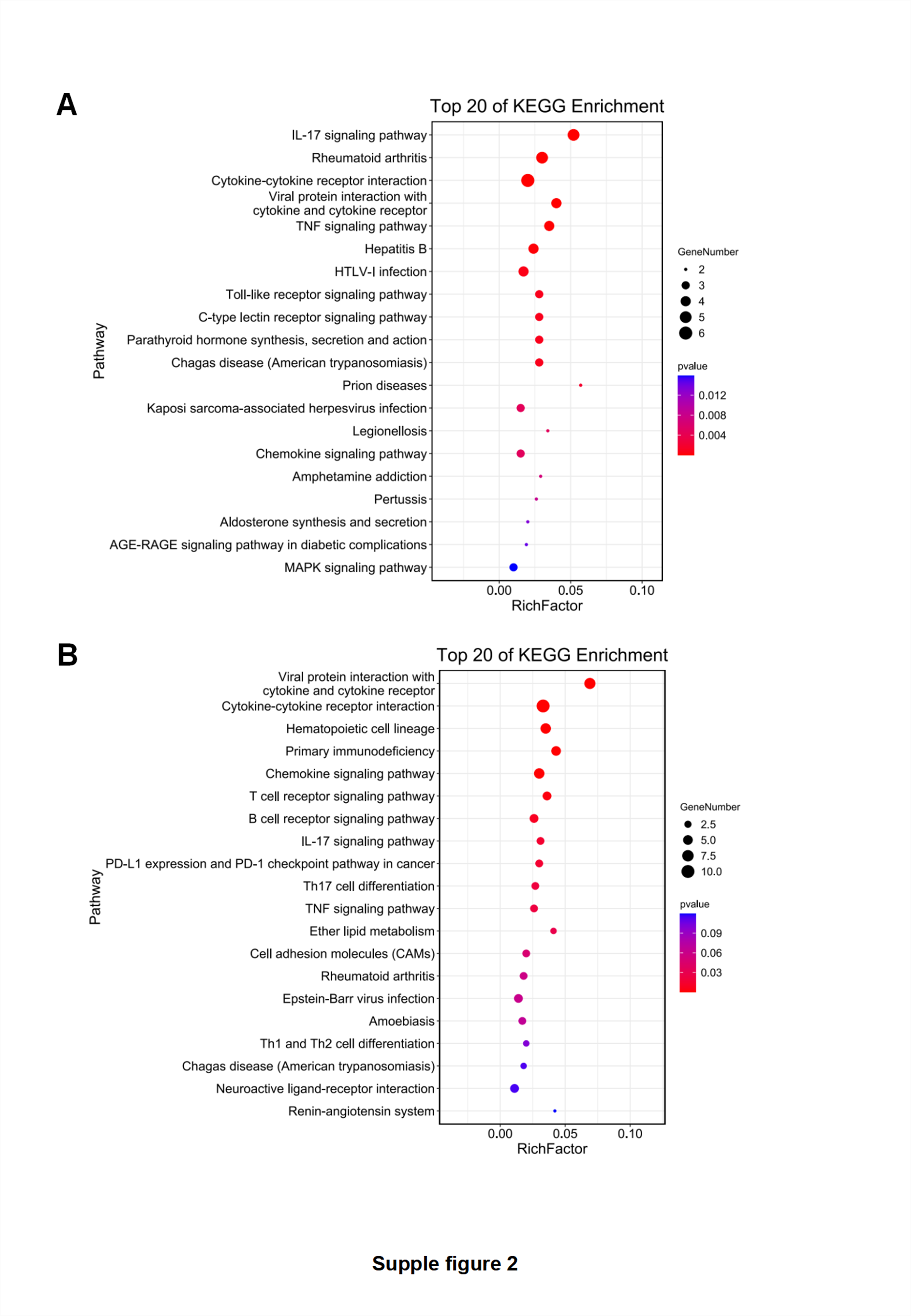

Supplement: Supplementary file 1 [file DataSheet1.ZIP › supple. Image and table/supple figure 2.tif]
